# Unusual OCT findings in a patient with *CABP4*-associated cone–rod synaptic disorder

**DOI:** 10.1007/s10633-023-09961-8

**Published:** 2024-01-11

**Authors:** Jit Kai Tan, Gavin Arno, Dragana Josifova, Moin D. Mohamed, Omar A. Mahroo

**Affiliations:** 1 Department of Ophthalmology, Guy’s & St Thomas’ NHS Foundation Trust, St Thomas’ Hospital, Westminster Bridge Road, London, England; 2 Institute of Ophthalmology, University College London, Bath Street, London, England; 3 Retinal and Genetics Services, Moorfields Eye Hospital, City Road, London, England; 4 Department of Genetics, Guy’s & St Thomas’ NHS Foundation Trust, Guy’s Hospital, London, England; 5 Section of Ophthalmology, King’s College London, St Thomas’ Hospital Campus, Westminster Bridge Road, London, England

**Keywords:** Retina, Photoreceptors, Congenital stationary night blindness, Cone dysfunction

## Abstract

**Purpose:**

Bi-allelic variants in *CABP4* are associated with congenital cone–rod synaptic disorder, which has also been classified, electrophysiologically, as incomplete congenital stationary night blindness (iCSNB). We describe clinical findings in a patient who demonstrated an unusual macular optical coherence tomography (OCT) phenotype, not previously reported in this condition.

**Methods:**

Our patient underwent multimodal retinal imaging, international standard full-field ERG testing and whole genome sequencing.

**Results:**

The patient was a 60-year-old woman with non-progressive visual impairment since birth, nystagmus and preference for dim lighting. Clinical fundus examination was unremarkable. OCT imaging revealed a hypo-reflective zone under an elevated fovea in both eyes. ERGs showed an electronegative DA10 response, with severely abnormal light-adapted responses. Whole genome sequencing revealed homozygosity for a known pathogenic variant in *CABP4*. No variants were found in other genes that could explain the patient’s phenotype.

**Conclusions:**

OCT findings of foveal elevation and an underlying hypo-reflective zone are novel in this condition. Whilst the clinical history was similar to achromatopsia and other cone dysfunction syndromes, ERG findings suggested disease associated with *CACNA1F* or *CABP4*. As *CACNA1F* is X-linked, *CABP4* was more likely, and confirmed on genetic testing. The patient saw better in dim light, confirming that night blindness is not a feature of *CABP4*-associated disease. Our case highlights the value of ERGs in discriminating between causes of cone dysfunction, and extends the range of retinal imaging phenotypes reported in this disorder.

## Background

Electroretinographically, congenital stationary night blindness (CSNB) has been classically divided into “Riggs-type” [1] (this rarer type entails reduced dark-adapted a-waves, indicating impaired rod phototransduction) and “Schubert–Bornschein-type” CSNB [2] (where the a-wave is of normal size, but the b-wave is selectively reduced). Miyake et al. [3] showed that the latter type could itself be divided into two subtypes, termed “complete” and “incomplete” CSNB, and proposed that they had different pathogenic mechanisms, which was proven true in subsequent decades, with different groups of genes being associated with each subtype.

In patients with complete CSNB (cCSNB, also termed CSNB1), there is selective loss of ON bipolar cell signals; the DA 0.01 ERG is usually completely abolished, and the LA 3 ERG shows a broadened a-wave trough with a sharply rising b-wave [3–6]. Genes associated with cCSNB include *NYX* (OMIM*300278), *TRPM1* (OMIM*603576), *GRM6* (OMIM*604096), *GPR179* (OMIM*614515) and *LRIT3* (OMIM*615004) [4, 6]. In incomplete CSNB (iCSNB, also termed CSNB2), the site of impairment is on the presynaptic side of the photoreceptor to bipolar cell synapse, and there is consequent attenuation of both ON and OFF bipolar cell signals. The DA 0.01 ERG is usually incompletely abolished, and the light-adapted ERGs are significantly more attenuated than in cCSNB; the LA 30 Hz flicker often shows a notched or bifid peak [3–6]. The genes classically associated with iCSNB are *CACNA1F* (OMIM*300110), which is on the X-chromosome, and, later, *CABP4* (OMIM*608965), where disease is associated with autosomal recessive inheritance [4, 6–8]. Whilst cCSNB invariably entails symptoms of night blindness, patients with iCSNB do not always complain of night blindness and sometimes symptoms of photophobia can be more pronounced. Hence it has been suggested that iCSNB can be a misnomer, particularly in the case of *CABP4*-associated disease, where night blindness is seldom, if ever, reported. For *CABP4*-associated disease, it has been suggested that “congenital cone–rod synaptic disorder” (OMIM #610427) should be used [8–10] or “*CABP4*-related retinal dystrophy” [11] or “*CABP4*-related disease” [10].

In the above conditions, clinical examination and retinal imaging findings tend to be largely unremarkable other than changes associated with high myopia in some cases. Some abnormal findings on OCT have been reported. Herein we describe a patient with molecularly proven *CABP4*-associated disease with abnormalities on spectral domain macular optical coherence tomography (OCT), namely foveal elevation over an underlying hypo-reflective zone, a novel finding in this condition.

## Case report

A middle-aged Caucasian woman was referred to our clinic with a history of lifelong non-progressive visual impairment since birth and a preference for dim lighting, for which she wore dark tinted contact lenses. She had a history of multiple squint surgeries and nystagmus. In her medical history, she had fibromyalgia, sleep apnoea and arthritis. Her medications were pregabalin and omeprazole. She had a sister with a similar eye condition, but no other affected family members.

**Fig. 1:**
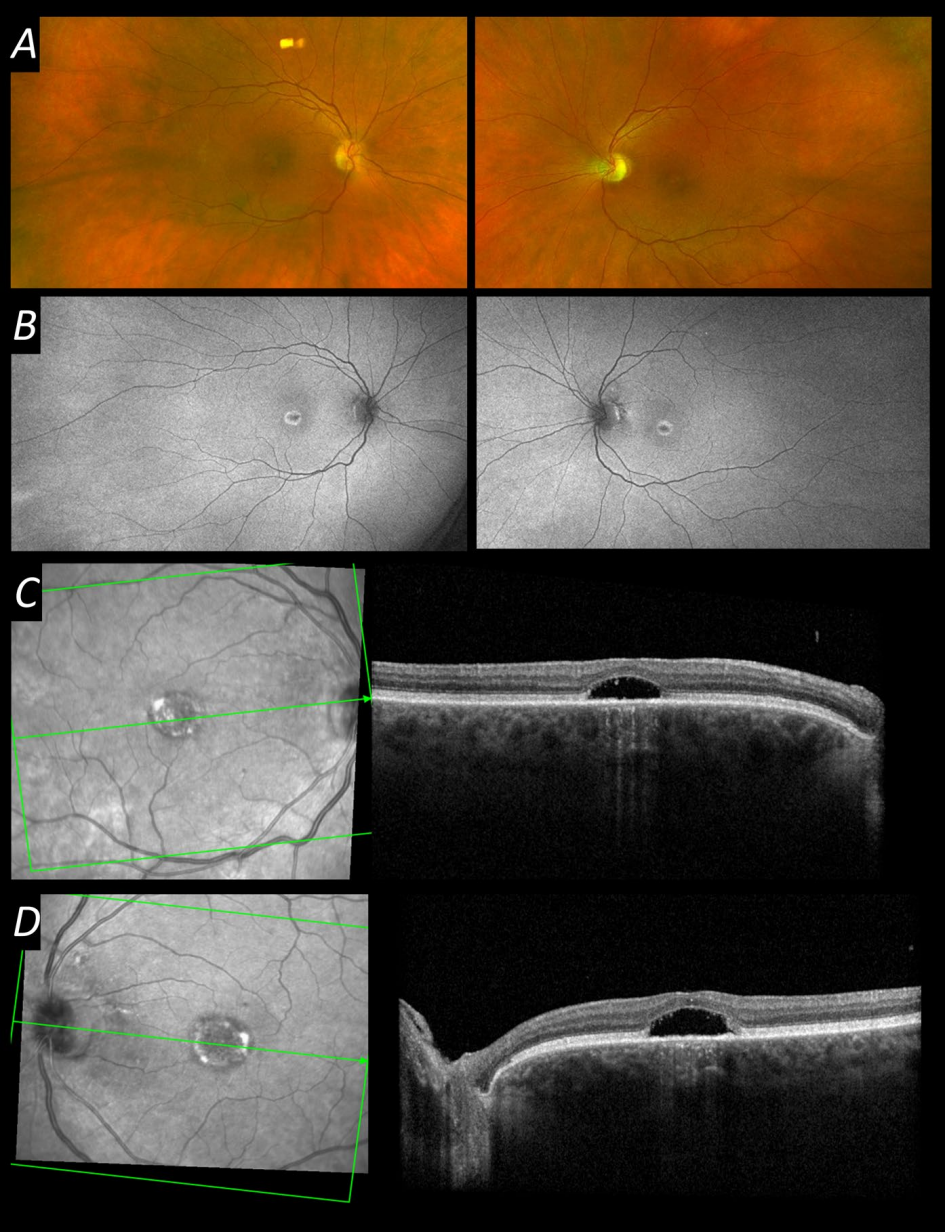
Multimodal retinal imaging from the patient aged 60. **A** Ultra-widefield pseudocolour fundus imaging (Optos, Dunfermline, UK) from both eyes. **B** Ultra-widefield green wavelength fundus autofluorescence imaging (Optos) from both eyes, showing abnormal central autofluorescence bilaterally. **C** Infrared reflectance image and macular OCT scan (Spectralis, Heidelberg, Germany) from the right eye. **D** Infrared reflectance image and macular OCT scan (Spectralis) from the left eye. Foveal elevation with subfoveal hyporeflectivity is seen on OCT in both eyes

**Fig. 2:**
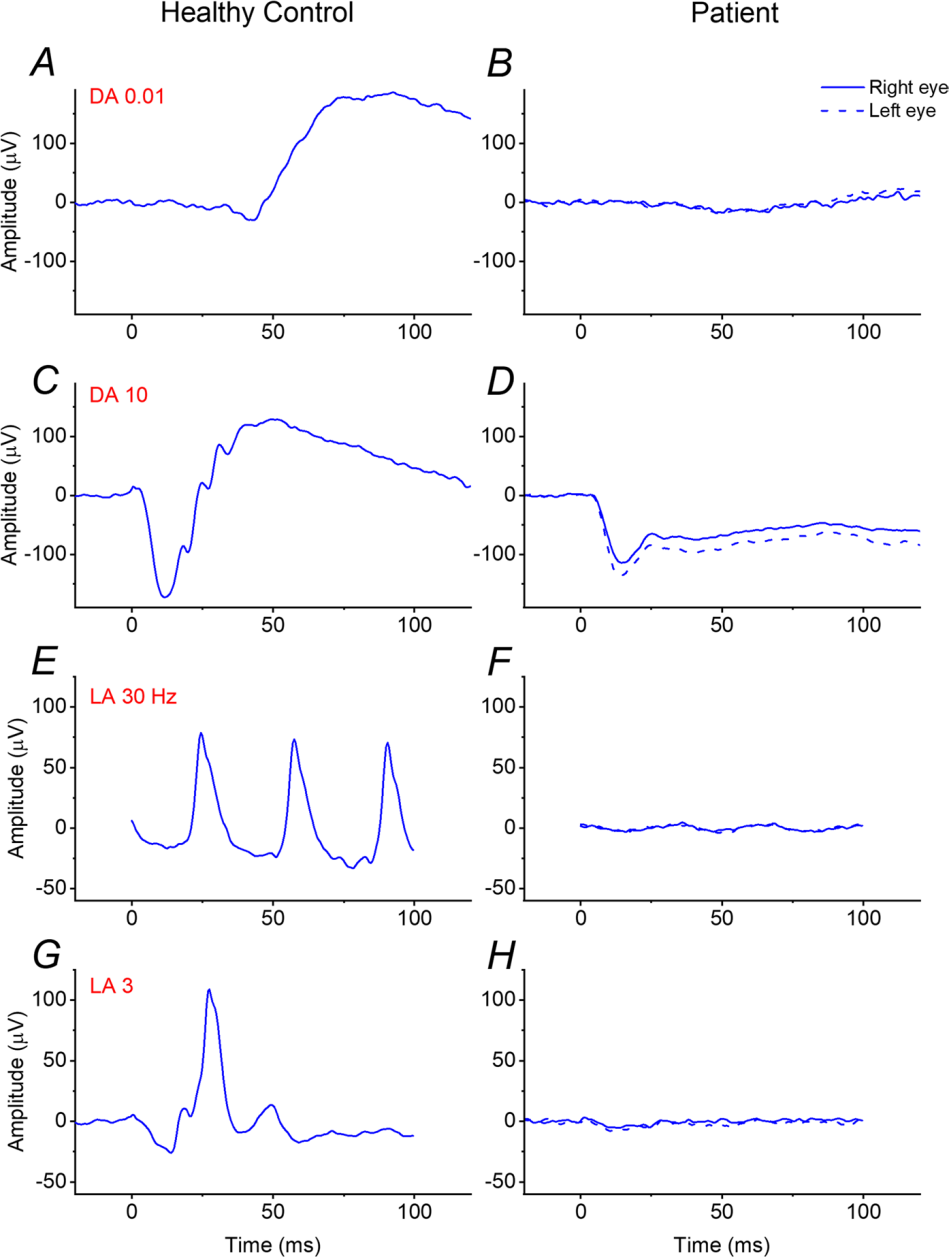
ISCEV standard full-field ERGs recorded from the patient aged 60. Responses were recorded with conductive fibre electrodes placed in the lower conjunctival fornix following pupil dilation. Left panels show responses from a healthy control; right panels show responses from the patient (dashed traces are from the left eye). **A**, **B** DA 0.01 responses are almost undetectable from the patient (**B**). **C**, **D** DA10 responses are electronegative from the patient (**D**), with the a-wave amplitude within normal limits. **E**, **F**, LA 30 Hz responses are severely attenuated in the patient (**F**). **G**, **H** LA 3 responses are also severely attenuated in the patient (**H**). DA 3 responses (not shown) were qualitatively similar to the DA 10 ERGs

**Fig. 3:**
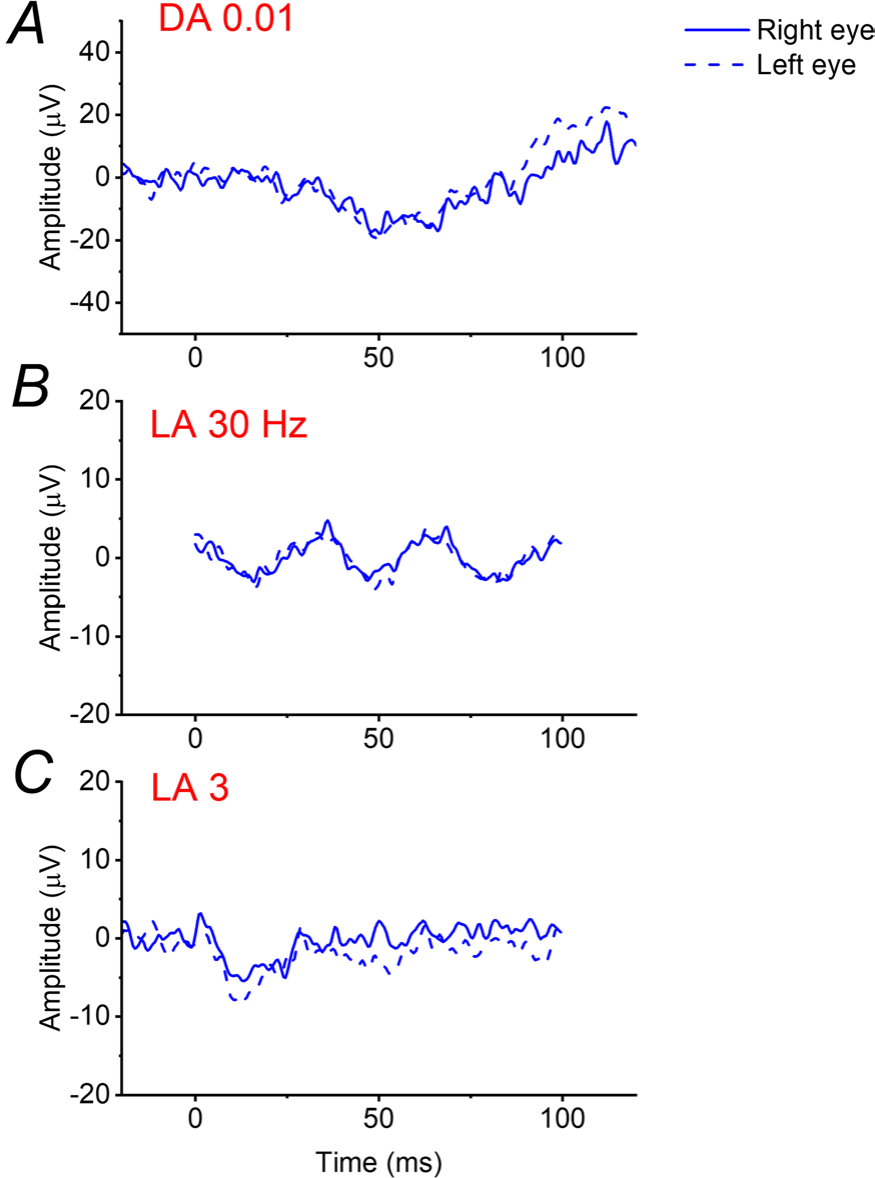
Selected ERGs reproduced from right panels of Fig. 2, with expanded *y*-axis scales. **A** DA 0.01 ERG showing a possible response just discernible at the expanded scale. **B** LA 30 Hz ERG. **C** LA 3 ERG showing a near-negative waveform in addition to severe attenuation

On examination, visual acuity was 20/200 in the right eye and left eye. She had a hypermetropic refractive error of + 3.50 D in each eye. Clinical anterior segment examination was unremarkable other than some mild lens opacity. Retinal examination was also unremarkable. Figure 1 shows multimodal retinal imaging findings. Autofluorescence imaging showed a central abnormality in both eyes, and OCT imaging revealed a hypo-reflective zone under an elevated fovea in both eyes. There also appeared to be a mild degree of foveal hypoplasia, with some persistence of inner retinal layers across the foveal centre. International standard ERG testing was performed (Fig. 2) [12]. Stimuli were delivered, and responses recorded, using the Diagnosys ColorDome with Espion software (Diagnosys LLC, Cambridge, UK); pupils were pharmacologically dilated, and conductive fibre electrodes (Unimed Electrode Supplies Limited, Farnham, Surrey, UK), placed in the lower conjunctival fornices, were used for ERG recording. Recordings showed an electronegative dark-adapted ERG, with severely abnormal light-adapted responses. Figure 3 shows some of the responses with a magnified y-axis scale, now showing a residual DA 0.01 response, as well as a near-negative waveform in the severely attenuated LA 3 ERG.

The patient underwent whole genome sequencing and was found to be homozygous for a pathogenic variant in *CABP4*. GRCh38 chr11:0.67457677C>T NM_145200.5:c.646C>T p.Arg216Ter. This variant was first reported in a Dutch family (two affected siblings) in 2009 [8].

## Discussion

The ERG findings pointed to impairment at the level of transmission at the synapse between photoreceptors and both ON and OFF bipolar cells. This, together with her history of lifelong, non-progressive visual impairment indicated a diagnosis of iCSNB or congenital cone–rod synaptic disorder. The more commonly associated gene with iCSNB is the X-linked *CACNA1F*, but variants in this gene cause disease in males, usually with high myopia (whilst our patient was female and hypermetropic). Our patient therefore was predicted to have *CABP4*-associated disease, and a homozygous pathogenic variant was indeed found in this gene.

There have been over 30 cases of *CABP4*-associated disease reported in the literature [7–9, 13–19]. Usually fundus findings are unremarkable, but some studies have reported foveal thinning, foveal hypoplasia or focal disruption of the ellipsoid zone [15, 16, 18]. Ages of patients in whom OCT findings have been reported in the literature range from under 1 year [18] to 16 years [16]. Our patient is significantly older and has an OCT phenotype not previously reported in this condition. Since she had no OCT imaging as a child or young adult, it is not known whether the structural abnormality represents an acquired change. The typical hyper-reflective deposits seen in vitelliform maculopathies were not seen in any of the OCT scans taken over multiple visits; and hence, her phenotype was not typical of adult vitelliform maculopathy.

Our patient’s preference for dim lighting supports the notion that night blindness is not a typical feature of *CABP4*-associated disease, as noted in several previous publications [8–11, 14]. Her ERGs showed near complete loss of the DA 0.01 response, highlighting that this does not occur solely in cCSNB. Indeed, the light-adapted responses may be more reliable in distinguishing between the two subtypes of Schubert–Bornschein CSNB (in terms of narrowing the differential diagnosis of likely genes).

*CABP4* encodes Ca^2+^-binding protein 4, which is photoreceptor-specific, localising to both rod and cone photoreceptor synapses, and necessary for their normal development and function [20–23]. It modulates the functional properties of photoreceptor L-type Ca^2+^ channels, interacting with the α1-subunit of the Ca_v_1.4 channel (encoded by *CACNA1F*). The important interactions between the two proteins likely help explain the similarity in ERG phenotype of diseases associated with variants in *CABP4* and *CACNA1F*. How synaptic abnormalities might relate to the OCT findings in our patient would be an intriguing subject of future research.

In summary, our case highlights the importance of electrophysiology in narrowing the genetic differential diagnosis and extends the range of OCT phenotypes associated with *CABP4*-associated disease.

## Declarations

### Statement on the welfare of animals

No animal experiments were done, so there is no reference to welfare of animals.
